# Microbial antigen in human milk: a natural vaccine?

**DOI:** 10.1038/s41385-022-00561-4

**Published:** 2022-08-27

**Authors:** Lieke W. J. van den Elsen, Tobias R. Kollmann, Valerie Verhasselt

**Affiliations:** 1 The University of Western Australia, School of Medicine, Perth, WA Australia; 2 Telethon Kids Institute, Perth, WA Australia

Infants are at high risk for infectious diseases, which account for over one third of deaths in children under 5 years of age^1^. Improving the effectiveness of vaccines, which have limited success in the first weeks of life, is important for better protection of this vulnerable population^2^. One overlooked avenue to help achieve long-term protection from infectious disease is newborn immunization through breast milk. Here we bring forward a new paradigm where imbibing microbial antigen-containing milk is akin to the process of vaccination, which captures the act of vaccine delivery. Bioactive agents in milk have potential to function as adjuvants, enhancing the infant’s immune response against an antigen. The infant’s immune response to the human milk containing microbial antigens with adjuvant molecules is the equivalent of immunization, resulting in less susceptibility to disease^3^.

Human milk is known to be the most potent way to prevent respiratory and gastro-intestinal infections^4^. This major impact is attributed to its high content in a wide array of anti-infective compounds such as maternal antibodies, lactoferrin and human milk oligosaccharides. These factors provide invaluable help for the developing immune system and can kill pathogens, inhibit their proliferation or prevent invasion of the mucosa of the breastfed child^4^. In addition to providing short-lived protection, there is evidence that microbial antigens in human milk may represent the optimal characteristics for infant mucosal immunization^4^. More in-depth understanding in this field is key for the prevention of infectious diseases and the development of age-tailored vaccination strategies.

## Evidence of infant vaccination through exposure to pathogen antigens in breast milk

The most striking observations of breast milk actively stimulating antigen-specific immune defences are related to maternal HIV infection (reviewed in^4^). More than 80% of children that are breastfed by HIV-positive mothers do not acquire HIV, despite ingesting HIV daily for several months to years. Importantly, these uninfected infants secrete HIV-specific IgG and IgA in the intestinal mucosa, demonstrating mucosal immune activation in response to HIV exposure through breast milk. In addition, almost half of them show HIV-specific IFN-γ responses in the blood, which persist after breastfeeding has ceased. These data remarkably illustrate that viral antigens in breast milk can stimulate both mucosal and systemic immunity in children, with an impact beyond the duration of breastfeeding. Supporting evidence for infant immunization through pathogen antigen shedding in breast milk is also found in maternal vaccination studies with live attenuated rubella virus. Rubella antigen is detected in the mother’s breast milk and infants born to rubella immunized mothers have rubella-specific IgG in serum and IgA in their nasopharyngeal secretions as well as virus-specific cellular immune reactivity in blood lymphocytes (reviewed in^4^). More recently, a study demonstrated development of mucosal immunity to SARS-CoV-2 in newborns breastfed by SARS-CoV-2 infected mothers, without any sign of infection in the offspring^5^. Parasitic antigens such as from *Schistosoma mansoni*^6^ and *Plasmodium falciparum*^7^ are also present in breast milk of infected women. While currently there is no data from birth cohorts on the possible immunization of children exposed to parasite antigen through breast milk, one study demonstrated better defence against *S. mansoni* in young adult mice when they had been nursed by *S. mansoni*-infected mothers^8^.

## How can antigen in breast milk effectively vaccinate children?

**Fig. 1:**
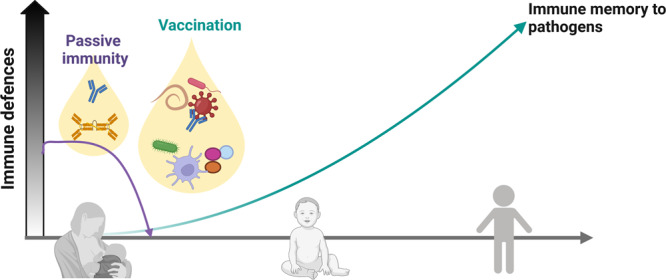
Breastmilk as a natural way of vaccinaton. Besides providing important but rapidly weaning passive immunity, breastmilk may actively provoke a long-lasting immune response by the transfer of low doses of microbial antigens in an infant-tailored vehicle. Breastmilk contains microbial antigens as a whole or (partially) pre-digested, presented by milk antigen-presenting cells or captured in extracellular vesicles or immune complexes. These antigens are delivered to the infant mucosa in conjunction with milk bioactive molecules and milk microbiota that can function as infant-tailored adjuvants. This may allow breastmilk to activate the neonatal immune system to induce protective, long-lasting immunity against pathogens. Image created with BioRender.

A few studies shed light on the possible ways breast milk vaccination may work and provide the optimal route to activate the newborn’s immune system (Fig. 1).

The very low amounts of antigens in human milk may fit the specific requirements for activation of the developing immune system. Neonates mount an efficient cytotoxic immune response to a viral dose 10,000 times lower than an adult, while higher doses are unable to activate an appropriate immune response^9^. The levels of *P. falciparum* antigen histidine-rich protein 2 and hepatitis B e antigen are 10-100-fold lower, and hepatitis B surface antigens are 30,000 times lower in human milk than in serum^7,10^. These low levels may be an important cue for the developing immune system of the neonate.

Exogenous proteins in human milk are pre-digested within the mammary gland. This process might be key for the generation of immunogenic peptides. As the newborn has only limited digestive abilities, predigesting pathogen-specific proteins in breast milk may be important for the newborn to generate a long-lasting and protective response^9^.

The presence of both maternal pathogen-specific antibodies and pathogens in breast milk highly suggests the presence of pathogen antigen-immune complexes. Antigen-IgG antibody immune complexes improve the transport of pathogen across the gut barrier using the neonatal Fc receptor and enhance the stimulation of effector immune responses by antigen-presenting cells^9^.

Antigen-presenting cells present in milk might play a role in the induction of a pathogen-specific immune response. Interestingly, the proportion of leucocytes in human milk increases upon maternal infection, which may increase pathogen-derived antigen presentation by milk antigen-presenting cells^9^. Human milk extracellular vesicles also express major histocompatibility complex molecules, which could contribute to the induction of antigen-specific immune responses in breastfed infants^9^.

Finally, microbes and microbial antigens in breast milk are surrounded by thousands of immune modulatory factors. The breast milk milieu contains molecules including antibodies and enzymes that can alter, weaken or reduce the viability of microbes present in the milk. This could lead to the generation of live attenuated pathogens that are fit to immunize the infant without infecting them. Among the bioactive compounds in breast milk are also potential strong adjuvants such as cytokines, the milk microbiota, soluble CD14 and Toll-like receptors.

The complex and dynamic composition of breast milk may have specifically been selected for and adapted to the newborn’s situation, in order to effectively promote immune defence upon microbial antigen transfer in the milk.

## Conclusion

Rather than merely a potential vehicle for transmission of disease, breast milk likely acts as a route that activates the neonatal immune system to mount a protective, long-lasting response. Very few studies have considered that pathogens and their antigens in human milk may immunise offspring. Further research is required to conclusively establish whether and how human milk from infected or vaccinated mothers can immunise their offspring.

Studies demonstrating active immunization of newborns through breastfeeding have important implications for maternal and child vaccination protocols. The knowledge can be used to develop infant-tailored, mucosal vaccines with an appropriate dose, adjuvant and form of the antigen for the developing immune system to react. In addition, maternal interventions to improve active immunization of newborns through breast milk, a physiological and needle-free route, are also in need of investigation. This includes improving antigen transfer into human milk and assessing storage and treatment conditions of expressed milk.

With most preventable deaths in under 5 years old children due to infections and in the light of the current COVID-19 pandemic, stimulation of immunity to antigens from pathogens through breastfeeding makes for an invaluable field to explore.
